# Electro-tactile stimulation of the posterior neck induces body anteropulsion during upright stance

**DOI:** 10.1007/s00221-018-5229-z

**Published:** 2018-03-16

**Authors:** A. M. De Nunzio, U. S. Yavuz, E. Martinez-Valdes, D. Farina, D. Falla

**Affiliations:** 1 Centre of Precision Rehabilitation for Spinal Pain (CPR Spine), School of Sport, Exercise and Rehabilitation Sciences, College of Life and Environmental Sciences, University of Birmingham, Birmingham, UK; 2 Institute of Applied Mechanics, University of Stuttgart, Stuttgart, Germany; 3 Department of Bioengineering, Imperial College London, Royal School of Mines, London, UK

**Keywords:** Electro-tactile stimulation, Postural control, Cutaneous mechanoreceptor afferences integration, Whole-body postural orientation

## Abstract

Sensory information conveyed along afferent fibers from muscle and joint proprioceptors play an important role in the control of posture and gait in humans. In particular, proprioceptive information from the neck is fundamental in supplying the central nervous system with information about the orientation and movement of the head relative to the rest of the body. The previous studies have confirmed that proprioceptive afferences originating from the neck region, evoked via muscle vibration, lead to strong body-orienting effects during static conditions (e.g., leaning of the body forwards or backwards, depending on location of vibration). However, it is not yet certain in humans, whether the somatosensory receptors located in the deep skin (cutaneous mechanoreceptors) have a substantive contribution to postural control, as vibratory stimulation encompasses the receptive field of all the somatosensory receptors from the skin to the muscles. The aim of this study was to investigate the postural effect of cutaneous mechanoreceptor afferences using electro-tactile stimulation applied to the neck. Ten healthy volunteers (8M, 2F) were evaluated. The average position of their centre of foot pressure (CoP) was acquired before, during, and after a subtle electro-tactile stimulation over their posterior neck (mean ± SD = 5.1 ± 2.3 mA at 100 Hz—140% of the perception threshold) during upright stance with their eyes closed. The electro-tactile stimulation led to a body-orienting effect with the subjects consistently leaning forward. An average shift of the CoP of 12.1 ± 11.9 mm (mean ± SD) was reported, which significantly (*p* < 0.05) differed from its average position under a control condition (no stimulation). These results indicate that cutaneous mechanoreceptive inflow from the neck is integrated to control stance. The findings are relevant for the exploitation of electro-tactile stimulation for rehabilitation interventions where induced anteropulsion of the body is desired.

## Introduction

Postural control is a skilled motor task based on processed activity of multimodal input integration (Maurer et al. 2006; Chiba et al. 2016) with the final aims of controlling the position of the center of body mass (CoM) allowing its ground projection to always fall inside the base of support (Winter et al. 2003) and keeping the desired posture based on the appropriate alignment of body segments relative to vertical (Kristjansson and Treleaven 2009). Controlling the centre of foot pressure (CoP) oscillations to counteract the torque generated by CoM motion is the final aim of achieving postural stabilisation. Sensorial aspects of the multimodal integrated nature of postural control have been widely studied on the grounds of the postural sway induced by stimulation of different sensory inputs (e.g., visual, vestibular, and proprioceptive) (Mergner et al. 2003; Chiba et al. 2016).

For example, visual inputs generate fast stabilisation of the extent of CoP oscillations (Wade and Jones 1997). Body sway induced by electric vestibular stimulation (galvanic stimulation) gives indications on how vestibular inputs are integrated and processed to manage postural control (Forbes et al. 2014). Proprioceptive input is used to control the body schema and to maintain the position of body segments according to the desired orientation (Vaugoyeau et al. 2008). In particular, in the neck region, the proprioceptive system is exceptionally developed, as segmental upper cervical muscles possess an unusually high density of muscle spindles (Peck et al. 1984). Therefore, using muscular vibration to stimulate proprioceptive afferences in the neck region prompts a strong body-orienting effect (forward body leaning) (Ivanenko et al. 2000; Courtine et al. 2007). Body anteropulsion results from the net effect of the nervous system compensation of the induced illusory lengthening of the stimulated muscles (illusory neck flexion) which, integrated with vestibular information of a vertically aligned head, corresponds to an illusory backward shift of the CoM (backward lean of the body) which might happen during a backward fall (Ivanenko et al. 2000; Courtine et al. 2007). This response highlights the important contribution of neck proprioception to the postural scheme used to control balance in humans (Roll et al. 1989; Massion 1992; Kavounoudias et al. 1999).

However, using high-frequency mechanical vibration (~ 100 Hz) to elicit a discharge of the segmental cervical muscle spindles can induce a concomitant activation of rapidly adapting mechanoreceptors (e.g., the Pacinian units) present in the vibrated skin area (Johansson et al. 1982) concealing their potential contribution to the noted postural effects. No previous studies have explored the contribution of the sole cutaneous mechanoreceptive units innervating the skin at the posterior aspect of the neck to the creation of the postural scheme and the control of body equilibrium.

We hypothesize that electro-tactile stimulation of the neck leads to body-orienting effects similar to those induced via muscular vibration, providing new insights into the contribution of tactile receptors in the control of posture. If confirmed, the obtained results may lead to new possibilities to use electro-tactile stimulation as a simple and effective way to develop new devices to enhance postural control in sensory-motor impaired subjects (e.g., elderly as well as people with neurological conditions) (Stolze et al. 2005; Horak 2006; Chiba et al. 2016) and to reduce the risk of falls.

Therefore, the aim of this study was to explore the involvement of the cutaneous mechanoreceptive units (tactile receptors) of the neck in the proprioceptive function of body schema formation (Ivanenko et al. 2000; De Nunzio et al. 2005; Courtine et al. 2007). To achieve this, we applied electro-tactile stimulation of the skin over the posterior region of the neck.

## Materials and methods

### Participants

An observational repeated-measures study was conducted on ten young healthy participants (8 males, 29.5 ± 5.5 years, mean ± SD). No participant reported any history of neurological, vestibular, or orthopaedic disorder. Informed written and oral consent was obtained from all participants prior to the intervention, which was performed according to the guidelines provided by the Declaration of Helsinki. The study was approved by the ethical committee of the University Medical Center, University of Göttingen, Germany.

### Electro-tactile stimulation

The tactile stimulation was generated controlling an isolated bipolar current stimulator (Digitimer DS5, Digitimer Ltd., Hertfordshire, UK) by an analogue voltage input. The stimulator translates the piloting signal in an isolated constant current stimulus which precisely replicates the input waveform. The analogue voltage input (stimulation signal) was generated programming a National Instruments analogue output board (PCI 6221, National Instruments Corporation, Austin, US) with LabVIEW-based custom-made software. The software controlled the stimulation signal and synchronised the timing of stimulation with the postural acquisition time (see “Task and procedures” session) towards a transistor–transistor logic (TTL) analogue signal.

The stimulation signal was a 1-ms biphasic symmetric sinusoid released at 100 Hz (one full-wave sinusoid each 0.01 s). The amplitude of the sinusoid was controlled via software and adjusted to reach 140% of the perception threshold of each subject to generate a distinct vibratory-like sensation falling within the commonly accepted range of useful frequencies for electro-tactile stimulation (Vallbo 1981; Szeto and Saunders 1982). The stimulus amplitude was limited to 10 mA.

### Task and procedures

**Fig. 1:**
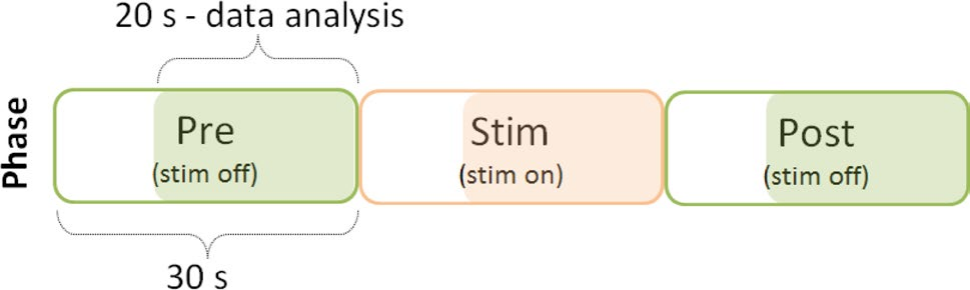
Experimental protocol. Graphical representation of the experimental protocol. The subject stood upright with their eyes closed. The acquisition, lasting 90 s in total, was divided into three phases (Pre, Stim, and Post) each of 30 s. The analysis on the acquired data was executed on the last 20 s of each phase to eliminate transients from the analysis

Participants were instructed to stand on a single force platform (Bertec, USA) with their eyes closed. Their feet were positioned in parallel at a distance of 10 cm. This position was marked for each subject on the platform to ensure consistency across trials (De Nunzio et al. 2008). Each postural acquisition lasted 90 s and was repeated three times. The acquisition trial was divided into 30 s consecutive phases: “Pre” (stimulation off), “Stim” (stimulation on), and “Post” (stimulation off) (see Fig. 1). The stimulus was delivered to two self-adhesive pre-gelled oval-shaped electrodes (4 × 6 cm, Krauth & Timmerman, Hamburg, Germany) placed over the spinous processes of the second and seventh cervical vertebra. Before electrode placement, the skin was gently abraded with abrasive paste (Nuprep®, Weaver and Company, US). The perception threshold was evaluated asking the subject to report when a subtle tactile sensation on the skin was felt, while the operator increased the stimulation current by 0.1 mA, every 3 s, starting from 0.5 mA.

### Data analysis and statistics

Mean position and standard deviation (SD) of the CoP oscillations, along the antero-posterior (A-P) and the medio-lateral (M-L) direction, were calculated as the main outcomes using the final 20 s of each acquisition phase to discard the transient effects induced by the electro-tactile stimulus (Fig. 1). While the CoP mean position gives an estimate of the extent of the postural net effect induced by the electro-tactile stimulation (e.g., leaning forward or backward), the SD of the CoP oscillations estimates the extent of body postural imbalance (amplitude of CoP oscillations) induced by electro-tactile stimulation (De Nunzio et al. 2008). As secondary outcomes, the time delays between (1) the electro-tactile stimulation onset and the display of a significant body forward leaning effect [Effect Delay, ED (s)], and (2) the stimulation stop and the recovery to the original postural position [Post-effect Delay (s), PD (s)] were calculated. ED and PD were extracted considering the time the CoP A-P component kept exceeding or falling below a “posture threshold” for more than 2 s. Before extracting the posture threshold, the average of the CoP A-P of the last 20 s of the “Pre” phase was subtracted from the entire CoP A-P track, to shift the analysed Pre phase of the CoP A-P to zero. The posture threshold was then calculated as the sum of the root mean square of the shifted CoP A-P track, executed on the last 20 s of the “Pre” phase, and twice the CoP A-P SD calculated over the same time window. The average of the three acquisition trials was calculated Fig. 3 for the analysis.

As data distributions were Gaussian (according to Shapiro–Wilk tests, *p* > 0.055 for all the analysed data), statistical evaluations were performed using a one-way repeated-measures analysis of variance (ANOVA) across the three acquisition phases (Pre, Stim, and Post) followed by Bonferroni corrected *t* tests when ANOVA was significant. Pearson r (ESr) was used to estimate the effect size and to evaluate possible correlation between analysed variables. ESr less than 0.19 was classified as “very weak”, 0.2–0.39 as “weak”, 0.4–0.59 as “moderate”, 0.6–0.79 as “strong”, and 0.8–1 as “very strong” (Evans 1996). 95% confidence interval (CI) for the mean difference was calculated. Significance was considered when *p* < 0.05. Statistical analysis was performed with SPSS software (IBM, Version 22). Post hoc analysis of the achieved power of the study was executed with the Software G*Power (version 3.1.9.2) (Faul et al. 2007) retrieving a power, 1 − *β* error probability, of 0.81 [*N* = 10, type I error probability *α* = 0.05, Cohen’s dz effect size = 1.01 (Cohen 1988)].

## Results

**Fig. 2:**
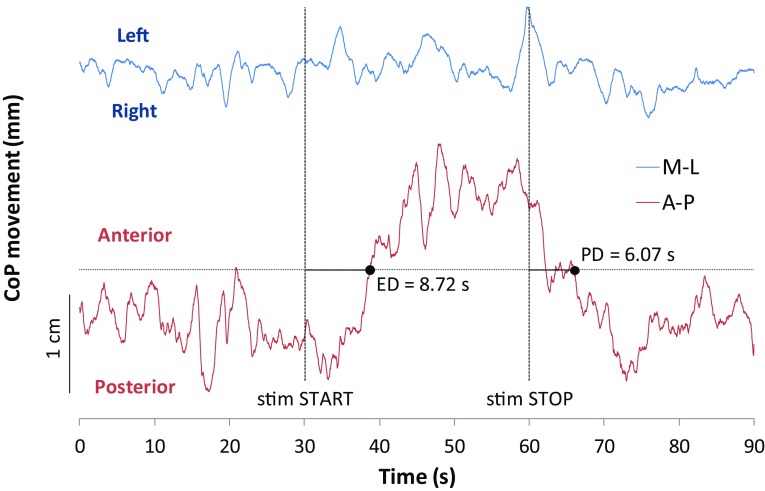
CoP movement along antero-posterior (A-P) and medio-lateral (M-L) direction. Oscillations of the center of foot pressure (CoP) of one subject reported along the entire duration of one trial (90 s). A-P and M-L axes are indicated in red and blue, respectively, along with the direction of the oscillations to show the effect of an anterior movement of the CoP during the stimulation phase (30 s = stim START − 60 s = stim STOP vertical dotted lines). The horizontal black dotted line depicts the posture threshold used to extract the Effect Delay (ED) and Post-effect Delay (PD) values, reported in seconds for the displayed track. No net effects can be appreciated along M-L axis

A mean ± SD stimulation peak of 5.14 ± 2.35 mA was used in the current study. Figure 2 reports a qualitative indication of the postural effects induced by an electro-tactile stimulation of the posterior aspect of the neck. The CoP position moved forward (along the A–P axis) during the stimulation phase with a delay in the anteropulsion effect (ED) of 8.72 s from the start of stimulation. A new stable forward body position was reached at almost 2 cm from the standing upright position at the Pre- phase. The original standing position was gained after 6.07 s from the end of the stimulation (PD value relative to a single trial). While a strong postural effect occurred along the A-P axis, the stimulation did not induce an increased oscillation or net movement of the CoP along the M-L axis.

**Fig. 3:**
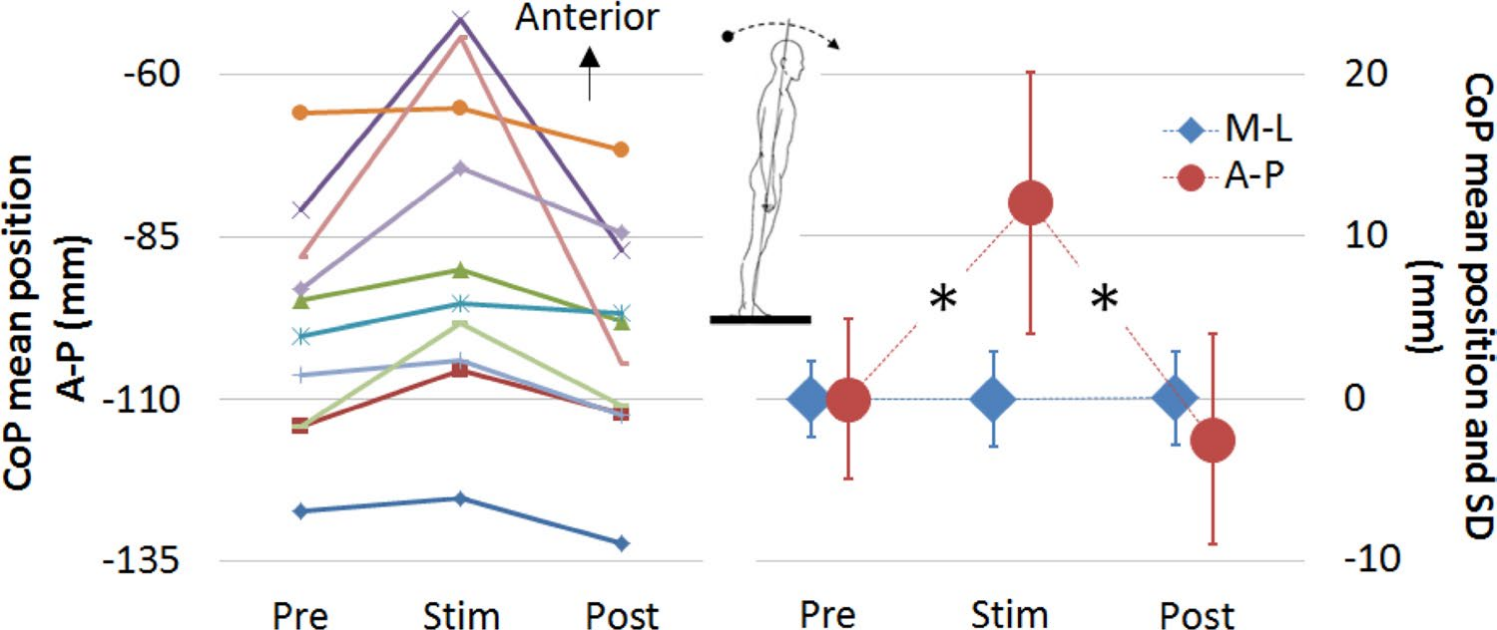
Mean and standard deviation (SD) of the center of foot pressure (CoP). Mean position of the CoP for each subject is reported on the left line plot for the three acquisition phases (Pre, Stim, and Post). The net effect of the stimulation along the antero-posterior (A-P, red markers) and medio-lateral (M-L, blue markers) axes is displayed on the right line plot. Mean values acquired at the Pre phase were subtracted from the Stim and Post phases for each subject to obtain the net effect of electro-tactile stimulation on body posture. The dot and diamond markers report the average position (mean of all the subjects) of the CoP along A-P and M-L, respectively. Error bars display the average (mean of all the subjects) SD of the CoP movement to report the extent of CoP A-P and M-L oscillations. A statistically significant difference (reported with an asterisk) is shown for the A-P CoP mean position between Pre vs Stim and Stim vs Post phases

Figure 3 shows the main outcomes of the study. The left plot Fig. 3 reports the mean A-P CoP position across the three acquisition phases (Pre, Stim, and Post) for each subject, indicating a consistent effect of a significant forward inclination of the body (forward leaning) induced by the stimulation. The right plot reports the net effect of the stimulation on A-P and M-L orientation of the body (leaning forward or backward and to the left side or right side, respectively) as the mean values for each subject, acquired during the Pre phase, were subtracted to the corresponding means extracted at the Stim and Post phases. Mean ± SD of the CoP A-P position was 12.08 ± 11.88 mm and − 2.5 ± 7.03 mm at Stim and Post, respectively (compared to Pre phase), indicating a net forward movement of the mean CoP position of approximately 1.2 cm, induced by the stimulation. The mean CoP A-P position during the Stim phase was statistically different from the Pre and Post phases (Fig. 3, *p* = 0.031, ESr = 0.88, − 23.1 < CI <− 1.05 Pre vs Stim and *p* = 0.046, ESr = 0.77, 0.22 < CI < 28.95 Stim vs Post). The mean CoP M-L position did not show any significant change (*p* > 0.05, 0.039 ± 2.92 and 0.077 ± 2.16 mm at Stim and Post, respectively) compared to the Pre phase.

The average SD of the CoP A-P and M-L oscillations (reported in Fig. 3 as error bars) did not show any statistically significant changes even if there was an increased extent of CoP oscillations during the Stim and Post phases for the A-P direction. The mean ± SD of the average CoP SD for A-P axis was 4.94 ± 1.93, 8.07 ± 4.88 and 6.49 ± 3.53 mm for Pre, Stim, and Post phases, respectively.

The average ED (9.05 ± 4.41 s) and PD (6.85 ± 3.13 s) were no significantly difference (*p* = 0.25) indicating a similar time delay to reach a different postural behaviour (forward lean) and to recovery from this position following the electro-tactile stimulation. Neither ED nor PD was correlated with the intensity of the electro-tactile stimulation (current peak in mA, *r* = 0.516, *p* = 0.126 for ED, and *r* = −0.187, *p* = 0.655 for PD). Moreover, neither ED or PD was correlated with the net body leaning effect induced by the stimulus, calculated as the average difference between CoP A-P mean position during Post vs Pre phases (*r* = −0.425, *p* = 0.22 for ED and *r* = −0.003, *p* = 0.993 for PD).

Since the statistically significant effect of forward leaning induced by stimulation could have been attributed to (1) the participants standing position or foot length, and (2) the intensity of the applied stimulation, Pearson’s correlation coefficient (*r*) was calculated (1) for the mean CoP A-P position between Pre vs Stim and Pre vs Post phases and (2) between the intensity of the electro-tactile stimulus (current peak, in mA) and the net leaning effect, as the average of the mean CoP A-P position at Stim minus Pre phase. All correlations were not statistically significant (i.*r* = 0.283, *p* = 0.426, for Pre vs Stim and *r* = −0.289, *p* = 0.416, for Pre vs Post; ii. *r* = 0.367, *p* = 0.295). Therefore, although the mean CoP A-P values at Pre phase depend on the foot length and starting position, there was no dependence between these individual characteristics and the postural effects induced via electro-tactile stimulation and a higher intensity of the applied electro-tactile stimulation did not account for a stronger leaning forward effect.

## Discussion

In healthy young subjects, a clear anteropulsion of the body is induced via subtle electro-tactile stimulation of the posterior aspect of the neck. The administered stimulus was meant to specifically activate the cutaneous mechanoreceptive units. Its relatively short duration provided a touch sensation and it was devised to minimally intrude on the vestibular system (Tashiro and Higashiyama 1981; Reynolds 2010; Volkening et al. 2014). The biphasic sinusoid was used as it generates less discomfort compared to a monophasic waveform and prevents charge accumulation, since the first pulse charge is discharged by the second pulse (Szeto and Saunders 1982). Moreover, biphasic pulse stimulation induces a sensory adaptation in as little as 15 min (Szeto and Lyman 1977) and the stimulation frequency (100 Hz) used was within the physiological discharge rate of the cutaneous mechanoreceptive units (Vallbo 1981) and within the range of useful frequencies for electro-tactile stimulation for sensory substitution in rehabilitation (Szeto and Saunders 1982). Even long exposure to this type of stimuli (10 h per day over 2 weeks) is safe, producing only mild and transient skin reddening (Szeto and Saunders 1982). The spatio-temporal characteristics of the induced postural effect (forward leaning) suggest that the administered electro-tactile stimulus mainly affected the cutaneous mechanoreceptive units and unlikely the muscle spindle afferences from the dorsal neck muscles. Indeed, electro-tactile stimulation of the neck cutaneous receptors induced a short forward sway (1.2 ± 1.1 cm) with a long effect delay (9.05 ± 4.41 s). In contrast, vibration of the dorsal neck muscles and, therefore, stimulation of the muscle spindles induce stronger “spatial” effects leading to an average of 5.6 cm of forward CoP sway [2.8 cm of SD, (Ivanenko et al. 2000)] and to an average effect delay as short as 1–2 s [visually appraised from (Ivanenko et al. 1999)].

This study is the first showing such a postural effect arising from the stimulation of the cutaneous mechanoreceptors of the neck. The effect was consistent across all participants and it has a clear direction along the antero-posterior axes of the body. However, as the stimulation was delivered with large electrodes, likely stimulating symmetrically over the left side and right side of the neck, the possibility for net postural effects along the medio-lateral axis might be discarded. The previous studies on cutaneous mechanoreceptor stimulation have only explored the postural stabilising effect of a noise signal transmitted to the skin via mechano-tactile or electro-tactile stimulation at lower limb sites (Priplata et al. 2003; Magalhaes and Kohn 2012, 2014). These studies explored the postural effects of a mechanism known as stochastic resonance which enhances the sensory detection ability, such that the receptor membrane potential fluctuates closer to the sensory threshold (McDonnell and Ward 2011).

As the stimulation area was in close proximity to the vestibular system, we cannot completely disregard the contribution of vestibular afferences to the presented results. However, the characteristics of the electro-tactile stimulus used in this study (1-ms biphasic symmetric sinusoid released at 100 Hz) are distinctive of mechanoreceptor stimulation and thus minimally intrude on the vestibular system (Johansson and Vallbo 1983). Galvanic vestibular stimulation, for example, is obtained using constant current stimuli delivered through the mastoid process or white noise-type stimuli, which has been used to induce postural effects (Reynolds 2010; Volkening et al. 2014). Moreover, evoked reflex responses can result from low (mechanical perturbation: 0–4 Hz) or high (electrical stimulation: 0–75 Hz) bandwidth stimulations (Forbes et al. 2014) which are still far from the stimulation frequency used in this study (100 Hz).

Involuntary body anteropulsion as a result of cutaneous mechanoreceptors stimulation shows how exteroceptive information, involved in the perception of the neck movement, is integrated to control whole-body orientation, to monitor the resultant position of the CoM and its projection on the base of support (Winter et al. 1996). The possibility of inducing a whole-body inclination effect, as induced via vibratory stimulation (Ivanenko et al. 2000), can be explained considering that neck tactile second-order afferences from cutaneous mechanoreceptors establish connection with the ventral posterior lateral nucleus of the thalamus to subsequently project to the primary somatosensory cortex from which the posterior parietal cortex (secondary somatosensory cortex level at area 5) is reached (Felleman and Van Essen 1991). The higher order somatosensory cortex, processes tactile information and uses proprioceptive signals for internal representations of integrated postures of the limbs (Mountcastle 1997; Kandel 2013). Besides, discharge of cutaneous mechanoreceptors at joint static position and movement, especially from the fast and slow adapting type II fibers (e.g. Pacinian corpuscle and Ruffini ending, respectively), indicates the involvement of such receptors in proprioceptive functions beyond their exteroceptive main purpose (Johansson and Vallbo 1983). The body anteropulsion induced by the electro-tactile stimulation of the neck can originate from the same compensatory mechanisms displayed during muscular vibration as net postural compensatory movement, induced by the change in the body representation, can be congruent with a backward CoM movement (as when leaning or falling backwards) (Ivanenko et al. 2000).

## Methodological considerations and future directions

The results obtained in this study provide useful indications for the development of innovative assistive devices based on surface electro-tactile stimulation to prevent falls in elderly people, people with sensory-motor impairment, or balance disorders (Melzer et al. 2004; Stolze et al. 2005; Cozart and Cesario 2009; Delval et al. 2014). Electro-tactile stimulation to the posterior aspect of the neck might be used to induce forward leaning of the body towards a safer standing position as optimisation of postural control, induced via motor learning, leads to a forward shift of the CoP (Tarantola et al. 1997). The safer postural set, reached with electro-tactile stimulation, has the potential to reduce fall incidence in elderly and people with neuromuscular disorders (Laughton et al. 2003; Melzer et al. 2004; Robinson et al. 2005; Whitney et al. 2006; Morrison et al. 2012). Moreover, electro-tactile stimulation induces involuntary postural effects (e.g., whole-body leaning forward) without disrupting postural control (as indicated by the non-significant changes in the SD of the CoP). Therefore, it could be used as a rehabilitation approach to actively engage people in coping with induced oscillations of the body. The forward as well as the slightly backward leaning position, reached during the Stim and Post phases, respectively, represents a more challenging condition (Schieppati et al. 1994), which correlates with an increased (although statistically non-significant) SD of the CoP oscillations. Body leaning occurs during transition movements (e.g., passing from standing to walking) when the CoM and the CoP move forward towards the limit of stability (before taking a step) (McCollum and Leen 1989; Schieppati et al. 1994) and represents a relevant motor task with a higher chance of falls (Delval et al. 2014; Faraldo-Garcia et al. 2016).

Future studies testing the body-orienting effects of the electro-tactile stimulation on prone-to-fall elders would overcome the limitations on the transferability of the results from the present investigation, since this study involved a small sample of young healthy participants. Surface electromyography of the neck extensor muscles could also be acquired in future studies to understand how the neck muscles behave during the induced forward lean.

## Conclusion

The current study reports, for the first time, the postural effect induced via electro-tactile stimulation of the posterior aspect of the neck. Cutaneous mechanoreceptor afferences from the neck induce a forward leaning posture and, as a consequence, a safer postural set. These results could be exploited to develop innovative assistive devices to improve motor control during posture and possibly decrease fall occurrence in the elderly and people with neuromuscular disorders.

## Abbreviations

CoM: Center of body mass
CoP: Center of foot pressure
TTL: Transistor–transistor logic
SD: Standard deviation
A-P: Antero-posterior direction
M-L: Medio-lateral direction
ESr: Pearson *r*
CI: Confidence interval
